# Stem cell-based combinatorial therapies for spinal cord injury: a narrative review of current research and future directions

**DOI:** 10.1097/MS9.0000000000001034

**Published:** 2023-07-03

**Authors:** Nicholas Aderinto, Muili Opeyemi Abdulbasit, Deji Olatunji

**Affiliations:** a Department of Medicine and Surgery, Ladoke Akintola University of Technology, Ogbomoso; b Department of Medicine and Surgery, University of Ilorin, Ilorin, Nigeria

**Keywords:** combinatorial therapies, spinal cord injury (SCI), stem cells

## Abstract

Spinal cord injury (SCI) is a devastating condition that can result in lifelong disability. Despite significant progress in SCI research, current treatments only offer limited functional recovery. Stem cell-based combinatorial therapies have emerged promising to enhance neural repair and regeneration after SCI. Combining stem cells with growth factors, biomaterials, and other therapeutic agents can improve outcomes by providing a multifaceted approach to neural repair. However, several challenges must be addressed before these therapies can be widely adopted in clinical practice. Standardisation of stem cell isolation, characterisation, and production protocols ensures consistency and safety in clinical trials. Developing appropriate animal models that accurately mimic human SCI is crucial for successfully translating these therapies. Additionally, optimal delivery methods and biomaterials that support the survival and integration of stem cells into injured tissue must be identified. Despite these challenges, stem cell-based combinatorial therapies for SCI hold great promise. Innovative approaches such as gene editing and the use of neural tissue engineering may further enhance the efficacy of these therapies. Further research and development in this area are critical to advancing the field and providing effective therapies for SCI patients. This paper discusses the current evidence and challenges from the literature on the potential of stem cell-based combinatorial therapies for SCI.

## Introduction

Highlights

- Stem cell-based combinatorial therapies hold significant potential for improving functional recovery in individuals with spinal cord injury. Combining stem cell therapy with other therapeutic approaches, such as biomaterial scaffolds and growth factors, can enhance the regenerative capacity of the injured spinal cord.
- Tailoring the therapy to individual patient characteristics, including the type and severity of the injury, is crucial for optimising outcomes and maximising functional recovery.
- Successful translation of these therapies into clinical practice has the potential to significantly improve the lives of individuals with spinal cord injury.

Spinal cord injury (SCI) represents a critical health concern due to its profound impact on individuals, their families, and society as a whole^1^. SCI often result in significant physical, sensory, and neurological impairments, leading to long-term disabilities and decreased quality of life for affected individuals^1^. SCI is a global health challenge of great significance, with varying prevalence and incidence rates across different regions^1^. While the global prevalence of SCI remains uncertain, the estimated annual incidence ranges from 40 to 80 cases per million population^1^. Recent studies have shed light on the prevalence and incidence rates of SCI in various regions. For instance, a study conducted in the Veneto Region reported a prevalence of 0.026% in a population of 4.9 million, identifying 1303 SCI cases from 2011 to 2020^2^. Another study in Nigeria documented 99 incidents of traumatic SCI, with a male predominance and complete tetraplegia involving the cervical spine being the most common clinical pattern^3^. A comprehensive review of 29 articles from seven countries from the Middle East and Africa revealed that the annual incidence of traumatic SCI per million was 23.24, with males accounting for 77% of the cases^4^. Despite the rising incidence of traumatic SCI, the global survey reports inadequate data on its prevalence worldwide. Comparable incidence data are only available for North America, Western Europe, and Australia^5^. Developing countries report higher 1-year mortality rates for traumatic SCI, with sub-Saharan Africa having the highest reported incidence of violence-related traumatic SCI worldwide^6^. Fatal outcomes for spinal injuries within a year are likely in some countries in sub-Saharan Africa^6^. Nevertheless, traumatic SCI survivability is better in developed countries, particularly for tetraplegia^7^.

SCIs constitute a significant global health concern resulting from various causes that vary by region. In North America, incidents of violence and self-harm, such as those involving firearms, are more prevalent, while motor vehicle accidents are the primary cause of traumatic SCI^8^. In contrast, falls from trees and rooftops cause SCI in Southern Asia and Oceania^9^. In some African countries, including Ethiopia, Ghana, Nigeria, Senegal, Sierra Leone, South Africa, and Zimbabwe, SCI is caused by a variety of factors such as motor vehicle accidents, falls from heights, violence, tunnel collapses in illegal mining, tuberculosis, malignant diseases, and HIV^6^. The non-traumatic SCI group in these countries had a higher average age than the traumatic group. In China, falls from heights were the primary cause of SCI, accounting for 30.8% of cases, followed by traffic accidents at 27.6% and low falls at 25.1%^10^. These various etiologies of SCI underscore the need for region-specific prevention strategies.

SCIs can be primary or secondary, with primary injuries resulting from mechanical disruptions or penetrating trauma, while secondary injuries arise from arterial disruption or hypoperfusion due to shock, exacerbating damage^11^. SCIs present with varying clinical manifestations depending on the affected spinal cord region, leading to different syndrome types. Management involves prompt intervention, adhering to guidelines like cervical spine immobilisation and logroll manoeuvre for transfers^12^. Circulatory support may be needed for cervical and upper thoracic injuries. Emerging therapies such as riluzole, minocycline, hypothermia, Magnesium chloride, exoskeletons, and stem cells hold promise for improved outcomes in SCI^13^.

The potential of stem cell therapy for the management of SCI is significant. Combinatorial therapies that employ multiple treatments or approaches for SCI management, including physical therapy, medication, surgery, stem cell transplantation, and electrical stimulation, are being explored to promote neural regeneration, reduce inflammation, and enhance functional outcomes in individuals with SCI^14^. This approach is promising and may offer better outcomes than single-modality treatments alone. In this narrative review, we aim to provide a comprehensive summary of current research on stem cell-based combinatorial therapies for SCI and discuss future directions for this exciting field specifically focusing on embryonic, induced pluripotent, and adult stem cells in combination with physical, gene and biomaterial therapy. We hope to stimulate further interest and research in this field by examining the latest advances and promising strategies.

## Methodology

To identify relevant studies published from January 2000 to April 2023, we conducted a thorough search of electronic databases, including PubMed, Embase, and Cochrane Library, using specific search terms such as “stem cells,” “spinal cord injury,” “combinatorial therapy,” “neural stem cells,” “mesenchymal stem cells,” “Schwann cells,” “physical therapy,” “medication,” “surgery,” “electrical stimulation,” “neuro-regeneration,” “inflammation,” and “functional outcomes.” In addition, we manually searched the reference lists of retrieved articles to identify additional studies. We included articles written in English reporting on stem cell-based combinatorial therapies for SCI in animal models and human subjects and reviews, excluding letters, editorials, and case reports. To ensure the accuracy and reliability of the data, two independent reviewers conducted the data extraction process, and any discrepancies were resolved through mutual discussion and consensus. We used the Cochrane Risk of Bias tool for animal studies and the Cochrane Collaboration tool for assessing the risk of bias in randomised clinical trials to evaluate the quality of the studies included in the review. Finally, the extracted data were synthesised and summarised narratively.

Our search identified 148 articles through electronic search; an additional 15 were identified through manual search of reference lists, resulting in 163 articles. After removing duplicates and screening for eligibility based on the inclusion and exclusion criteria, 79 articles were included in this review. By synthesising the data from these studies, we aim to provide an up-to-date and comprehensive understanding of the potential of stem cell-based combinatorial therapies for SCI and stimulate further interest and research in this exciting field.

## Types of stem cells used in combinatorial therapies

The therapeutic potential of stem cells in SCI has garnered significant interest due to their unique characteristics, including self-renewal and differentiation into various cell types. Combinatorial therapies utilising different types of stem cells, such as embryonic stem cells (ESCs), induced pluripotent stem cells (iPSCs), neural stem cells (NSCs), mesenchymal stem cells (MSCs), and Schwann cells, in conjunction with physical therapy, medication, surgery, and electrical stimulation have shown promise in promoting neural regeneration, reducing inflammation, and enhancing functional outcomes. While each stem cell type possesses unique therapeutic properties, a comprehensive understanding of their mechanisms of action is necessary for developing effective and personalised therapies for SCI (Table 1).

**TABLE 1:** Summary of stem cell types used in SCI research: properties, advantages, and limitations.

Stem cell type	Properties	Advantages	Limitations
Embryonic stem cells	Pluripotent, can differentiate into any cell type	High proliferative capacity, potential for large-scale production	Ethical concerns, risk of teratoma formation, immune rejection
Neural stem cells	Multipotent, can differentiate into neurons and glia	Intrinsic tropism for CNS, potential for endogenous repair	Limited availability, limited differentiation potential
Mesenchymal stem cells	Multipotent, can differentiate into mesodermal lineages	Immunomodulatory effects, anti-inflammatory properties, easy to obtain	Low differentiation potential, variability between donors, limited integration
Induced pluripotent stem cells	Pluripotent, can differentiate into any cell type	Personalised medicine, avoids ethical concerns	Potential for genetic abnormalities, risk of tumorigenesis, low differentiation efficiency
Olfactory ensheathing cells	Glial cells that support olfactory axons	Intrinsic tropism for CNS, promote axonal regeneration, myelination, and neuroprotection	Limited availability, low proliferation rate, variable results
Schwann cells	Glial cells that myelinate peripheral nerves	Promote axonal regeneration, myelination, and neuroprotection	Limited availability, require peripheral nerve biopsy, limited integration

CNS,Central Nervous System; SCI, spinal cord injury.

### Embryonic stem cells

ESCs have emerged as a promising avenue for regenerative medicine, owing to their exceptional plasticity and capacity to differentiate into any human cell type^15^. Dr. James Thomson’s pioneering work popularised the potential therapeutic use of ESCs, particularly for patients with motor or sensory function loss who have limited treatment options^16^. ESCs are derived from in-vitro fertilised embryos and can be collected at varying stages of development while preserving their potential for normal development^17^. These self-regulating cells can differentiate into any human cell type, although their ability to regenerate in utero is limited. In contrast to embryonal carcinoma, cells derived from teratocarcinoma, which were previously believed to be stem cells but proved fatal when implanted in adults, human ESCs sourced from the inner cell mass of the human blastocyst have a normal karyotype and can differentiate into any of the three embryonic germ layers at any point during cell culture^17^.

The differentiation of ESCs into neural cells is a complex process that poses challenges due to the potential development of unwanted cell types^18^. Studies have been investigating specific factors that can facilitate the differentiation of ESCs into desired cell types, such as oligodendrocytes and motoneurons. However, the safety of ESC transplantation remains a major concern, as teratoma formation has been reported in some cases^19^. Reducing the risk of tumour formation can be achieved by employing prolonged differentiation or genetic manipulation. Additionally, researchers are exploring using scaffolds made from cellular matrix proteins to support and release growth factors that aid cell survival and differentiation. Recent studies have demonstrated the potential of using ESC-derived motoneurons to integrate into the spinal cord, leading to functional recovery of lost motor function^20,21^.

Combining ESC transplantation with other treatments, such as physical therapy and pharmacological interventions, is being investigated as a potential treatment option for SCI and other neurological disorders^22^. For example, combining ESC transplantation with chondroitinase ABC, an enzyme that degrades the inhibitory extracellular matrix in SCI lesions, has shown promise in promoting axon regeneration and functional recovery in animal models^23^. Electrical stimulation combined with ESC transplantation has also been used to promote the differentiation of ESCs into motoneurons, leading to functional recovery of lost motor function in animal models of SCI^24^.

### Induced pluripotent stem cells

iPSCs are generated by reprogramming adult somatic cells, such as skin cells, into a pluripotent state akin to ESCs by introducing specific genes^25^. iPSCs can differentiate into any human cell type and hold substantial promise for treating SCI and other neurological disorders.

The discovery of iPSCs marked a significant milestone in regenerative medicine, as it resolved the ethical concerns associated with ESCs that use embryos^26^. Furthermore, iPSCs have the potential to provide personalised cell-based therapies, as they can be generated from a patient’s cells, minimising the risk of immune rejection^27^. Despite their potential, several challenges must be addressed before they can be used in clinical applications. One significant concern is the risk of tumorigenicity, as iPSCs can form tumours when implanted *in vivo* ^28^. Researchers are exploring ways to mitigate this risk by identifying specific factors that enhance the differentiation of iPSCs into desired cell types and developing safer delivery methods. Additionally, the efficiency of iPSC generation and genomic instability must be addressed before clinical use. Despite these challenges, iPSCs hold significant promise in treating SCI and other neurological disorders. These cells can differentiate into neural cells, critical for repairing damaged spinal cords. Researchers can manipulate iPSCs to express specific factors that promote differentiation into desired cell types, making them an attractive option for developing combinatorial therapies for SCI. With continued research and development, iPSCs have the potential to revolutionise regenerative medicine and significantly improve the lives of patients with SCI and other neurological conditions.

### Adult stem cells

Unlike ESCs, which have the potential to form tumours and raise ethical concerns, adult stem cells are derived from various tissues in the body and have a more limited ability to differentiate into other cell types^29^. However, adult stem cells can still differentiate into neural cells and have demonstrated therapeutic potential in animal models of SCI. Several types of adult stem cells have been investigated for SCI treatment, including MSCs, NSCs, and olfactory ensheathing cells (OECs).

MSCs, found in various tissues such as bone marrow and adipose tissue, possess immunomodulatory and anti-inflammatory properties, making them an attractive option for SCI treatment^30^. Furthermore, MSCs can differentiate into neural cells and promote axon growth in animal models of SCI. NSCs, found in the nervous system, can differentiate into different types of neural cells and have been shown to improve motor function and promote axon growth in animal models of SCI^31^. OECs, specialised cells found in the olfactory system, can promote axon growth and myelination in animal models of SCI^32^. In addition to their ability to differentiate into neural cells and promote axon growth, adult stem cells can have a paracrine effect, secreting various growth factors, cytokines, and chemokines that promote tissue repair and regeneration^33^. This effect is particularly important for MSCs, which secrete factors that can modulate the immune response and promote tissue repair. Although adult stem cells hold promise for SCI treatment, further research is needed to optimise their use. For example, more research is needed to identify the most effective delivery methods and dosages of adult stem cells. Additionally, more studies are needed to determine adult stem cell transplantation’s long-term safety and efficacy in human subjects. Nevertheless, adult stem cells remain a promising avenue for developing effective therapies for SCI.

Clinical trials evaluating the safety and effectiveness of adult stem cell therapy for SCI have demonstrated encouraging results. A phase I/II clinical trial examined autologous bone marrow-derived MSCs in patients with acute SCI and found that the treatment was well-tolerated and safe^34,35^. Moreover, the study found that MSC treatment patients had improved motor and sensory function compared with the control group. A phase II clinical trial, which investigated autologous bone marrow-derived MSCs in patients with chronic SCI, also demonstrated that the treatment was safe, well-tolerated, and significantly improved motor function^36^. Other clinical trials examining NSCs and OECs for SCI treatment have also demonstrated encouraging results. A phase I clinical trial that studied the use of autologous NSCs in patients with chronic SCI revealed that the treatment was safe, well-tolerated, and improved sensory function^36^.

## Combinatorial approaches involving stem cells

Combinatorial therapies that include stem cells have shown promise in preclinical studies for SCI. While stem cells alone have had limited success in achieving functional recovery after SCI, incorporating other therapeutic strategies, such as rehabilitation, growth factors, and biomaterials, has demonstrated potential in animal models^37^ (Table 2). These approaches can promote neural regeneration, modulate inflammation, and improve functional recovery after SCI.

**TABLE 2:** Summary of combinatorial approaches for spinal cord injury: components and effects on axonal regeneration, myelination, neuroprotection, and functional recovery.

Combinatorial approach	Components	Effects on spinal cord injury
Stem cells + growth factors	Neural stem cells + BDNF, NT-3, GDNF	Promote axonal regeneration, myelination, and functional recovery
Stem cells + scaffolds	Mesenchymal stem cells + chitosan, collagen, fibrin, hyaluronic acid	Provide mechanical support, promote cell survival and differentiation, and improve functional recovery
Stem cells + immune modulation	Induced pluripotent stem cells + regulatory T cells, cytokines, antibodies	Modulate immune response, reduce inflammation, and promote tissue repair
Stem cells + exosomes	Olfactory ensheathing cells + exosomes derived from MSCs or neural stem cells	Deliver trophic factors, promote axonal growth and myelination, and reduce apoptosis
Stem cells + electrical stimulation	Schwann cells + conductive polymers, electric fields	Enhance cell migration, promote myelination and axonal regeneration, and improve functional recovery
Stem cells + gene therapy	Neural stem cells + growth factors, transcription factors, CRISPR/Cas9	Enhance cell survival, differentiation, and integration, and promote tissue repair and functional recovery
Stem cells + physical therapy	Mesenchymal stem cells + locomotor training, range-of-motion exercises	Improve motor function, muscle strength, and quality of life

BDNF, brain-derived neurotrophic factor; GDNF, cell-derived neurotrophic factor; MSC, mesenchymal stem cell; NT-3, neurotrophin-3.

### Stem cells and biomaterials

Combining stem cells with biomaterials enhances their survival and integration into the injured tissue, leading to functional recovery. In addition, biomaterials provide physical support and a three-dimensional environment for stem cells, promoting their survival and differentiation into the desired cell types.

Hydrogels have gained attention as a biomaterial for use with stem cells due to their biocompatibility and ability to mimic the extracellular matrix of tissues. A study using a hyaluronic acid-based hydrogel to deliver human neural stem cells to a rat model of SCI demonstrated the potential of this approach to promote stem cell survival and differentiation, leading to functional improvement^38^. However, hydrogels have limited mechanical strength and can degrade over time, limiting their long-term effectiveness. Scaffolds, on the other hand, provide mechanical support and a structure for stem cells to grow on, allowing for the delivery of stem cells to the site of injury and promoting their survival and differentiation. A polycaprolactone scaffold was used to deliver mesenchymal stem cells to a rat model of SCI, resulting in functional improvement^39^. The design of scaffolds can also be tailored to have specific properties such as porosity and mechanical strength, which can be customised to meet the specific needs of the repaired tissue^40^. However, the use of scaffolds can be limited by the potential for immune rejection and the need for additional surgery to remove the scaffold after the repair process is complete.

Microcarriers, such as alginate microbeads, have also been studied for their ability to provide a three-dimensional environment for stem cells to proliferate^41^. They can be designed to have specific properties, such as size and surface charge, which can be tailored to the specific needs of the repaired tissue. However, the use of microcarriers can also be limited by the potential for immune rejection and the need for additional surgery to remove the microcarriers after the repair process is complete.

### Stem cells and gene therapy

The combination of gene therapy and stem cell transplantation has shown great potential for treating SCI. In this approach, genes are delivered to the injury site to promote stem cells’ survival and differentiation. A recent study used a lentiviral vector to deliver the gene encoding for glial cell line-derived neurotrophic factors to human neural stem cells before transplantation into a rat model of SCI^42^. This combinatorial therapy resulted in enhanced functional recovery in the rats. However, further research is needed to optimise the delivery and efficacy of gene therapy in combination with stem cells to treat SCI.

Genetically modified stem cells offer a promising avenue for developing effective SCI treatments. Researchers have been able to engineer neural stem cells and mesenchymal stem cells to express genes such as brain-derived neurotrophic factor (BDNF), neurotrophin-3, and glial cell-derived neurotrophic factor (GDNF), which are involved in nerve cell survival, differentiation, and repair^43^. By delivering these cells to the site of injury, researchers have stimulated the growth of new nerve cells and improved motor function in animal models of SCI. Despite the promising results, using genetically modified stem cells for SCI treatment faces important challenges. One of the most significant obstacles is optimising the delivery and efficacy of these cells to achieve consistent and durable results. Furthermore, the safety and potential risks associated with gene therapy must be rigorously evaluated to ensure that the benefits of this approach outweigh the potential harm. Nonetheless, these advances offer a promising avenue for the future of SCI treatment, and continued research in this field holds great potential for developing novel stem cell-based therapies.

### Stem cells and physical therapy

Physical therapy is essential in supporting the survival and differentiation of transplanted stem cells in animal models of neurological injury. By improving the microenvironment of the injury site, physical therapy creates a more supportive environment for the transplanted stem cells to integrate into the surrounding tissue^44^. Exercise and other physical therapies increase the secretion of growth factors and other molecules that promote the survival and differentiation of transplanted stem cells, enhancing their therapeutic potential for neurological injury^45^.

Stem cell transplantation complements physical therapy by providing a source of cells that can differentiate into cells needed for repair and regeneration. Stem cells also secrete growth factors and other molecules that promote the survival and differentiation of endogenous cells, enhancing the effects of physical therapy. In preclinical studies, the combination of stem cell transplantation and physical therapy has demonstrated greater functional recovery than either treatment alone^46,47^.

Stem cell transplantation can provide a source of cells for repair and regeneration, while physical therapy can create a more supportive microenvironment for the transplanted cells to survive and differentiate. Moreover, stem cells may secrete growth factors and other molecules that enhance the effects of physical therapy. Combination therapy may also reduce the risk of adverse effects associated with stem cell transplantation by optimising the therapeutic potential of the transplanted cells. Nonetheless, further research is required to optimise the timing and dosage of stem cell transplantation in combination with physical therapy to develop this approach for clinical use.

## Preclinical studies of stem cell-based combinatorial therapies for SCI

Preclinical studies have investigated the therapeutic potential of stem cell-based combinatorial therapies for treating SCI (Table 3). These studies have explored the use of stem cells in combination with various therapies, including physical therapy, pharmacotherapy, and gene therapy. Most of these investigations have focused on hindlimb and gait function in rodents, with only a limited number of studies performed on minipigs, felines, and non-human primates. The thoracic cord injury model and physical training, which includes hindlimb motion, have been used in many of these studies due to their advantages of being more easily assessed and having a higher survival rate, as well as the applicability to various types and severities of SCIs.

**TABLE 3:** Key preclinical studies on combinatorial therapies for SCI.

Author	Year	Modality	Result
Wang *et al*.^49^	2020	Matrigel as scaffold material	Improved functional repair and behavioural recovery
Mothe *et al*.^50^	2013	Hyaluronan-based hydrogel	Significant reduction in cavitation and sparing of perilesional host oligodendrocytes and neurons
Raynald *et al*.^51^	2019	Polypyrrole/polylactic acid (PPy/PLA) nanofibrous scaffold	Promote the functional recovery of the spinal cord
Park *et al*.^52^	2012	Matrigel	Improved functional recovery
Jiao *et al*.^53^	2017	Silk fibroin/alginates/glial cell line-derived neurotrophic factor (SF/AGs/GDNF) scaffold	Better therapeutic and repair effects
Wang *et al*.^54^	2017	Poly lactic-co-glycolic acid (PLGA)	Enhanced locomotor recovery, axon myelination and better protected neurons post SCI
Wang *et al*.^55^	2021	Nerve growth factor	Enhanced the motor function of hindlimbs
Keikhaei *et al*.^56^	2023	High-intensity interval training	Improved locomotor function
Shibata *et al*.^57^	2023	Rehabilitative Training	Significant improvement in motor functions
Chen *et al*.^58^	2008	Electro-acupuncture	Improved functional recovery
Ding *et al*.^59^	2009	Electro-acupuncture	Significant functional recovery

SCI, spinal cord injury.

### Preclinical stem cell therapies with biomaterial scaffolds

Studies utilising hydrogel blends and matrigel in conjunction with non-progenitor stem cells in rats have yielded promising results, including improved graft survival, behavioural recovery, and neuronal regeneration^48–50^. Other studies have explored the therapeutic benefits of combining neural-induced mesenchymal stem cells with matrigel and bone marrow stromal cells with a polypyrrole/polylactic acid nanofibrous scaffold^51,52^. These combination therapies successfully inhibited scar tissue formation, promoted axon regeneration, and facilitated functional recovery in animal models of SCI.

Recently, researchers have evaluated the therapeutic potential of a hybrid treatment system involving silk fibroin, alginates, and GDNF scaffolds with human umbilical cord mesenchymal stem cells (hUCMSCs) in rat models of SCI^53^. In another study, researchers explored the therapeutic potential of poly(lactic-co-glycolic acid) complexes inoculated with OECs. They observed improved locomotor recovery, axon myelination and enhanced neuroprotective and neuroregenerative abilities in treated rats^54^.

These preclinical studies demonstrate the potential of combination therapies utilising various stem cell types and biomaterial scaffolds in promoting functional recovery and neuronal regeneration in animal models of SCI. Further research is needed to optimise these combination therapies’ timing, dosage, and clinical applications.

### Preclinical stem cell therapies with neurotrophic factors and physical therapy

NSCs are commonly employed in combination with growth factors, including neural growth factor (NGF), GDNF, and BDNF, to assess their potential for neuroprotection and functional recovery in SCI. In a recent study, NGF-NSCs were transplanted into rats with SCI to evaluate their effects on hindlimb motor function and endogenous neurogenesis^55^. The results showed that NGF-NSCs significantly improved hindlimb motor function and promoted functional recovery by modulating the microenvironment and enhancing endogenous neurogenesis. Similarly, genetically modified human neural stem cells overexpressing BDNF (F3.BDNF) were shown to promote functional recovery in SCI rats, as evidenced by improved locomotor function.

Although studies investigating the combination of stem cell therapy with physical therapy are limited, a recent study evaluated whether neural progenitor cell grafts combined with rehabilitation can lead to functional recovery in chronic SCI^37^. In this study, rats underwent intensive rehabilitation in conjunction with stem cell therapy, significantly increasing neuroregeneration and functional recovery. Another recent study evaluated the combined treatment of high-intensity interval training with neural stem cell transplantation in rats with SCI, which improved locomotor functions and histological recovery^56^. These findings suggest that combining stem cell therapy with physical therapy may hold considerable potential as a therapeutic approach for SCI.

Functional recovery induced by rehabilitative training has been reported in many studies. Changes such as regeneration and reorganisation of spinal descending circuits, enforcement of synaptic function, axonal regeneration, exercise-dependent plasticity, and motor control improvements through restoring spinal inhibitory capacity, sensory-motor integration, and supra-spinal control have been demonstrated. Various neurotrophic factors, growth factors, and excitatory and inhibitory molecules have been suggested to be involved in these changes. A recent study by Shibata *et al.* ^57^ investigated whether combining clinical-grade human-induced pluripotent stem cell-derived neural stem/progenitor cells transplantation and rehabilitative training could enhance functional recovery in a rodent model of chronic SCI. The results showed that the combination therapy significantly promoted the survival rate and neuronal differentiation of transplanted human-induced pluripotent stem cell-derived neural stem/progenitor cells and increased the expression of the BDNF and neurotrophin-3 proteins in the spinal cord tissue. Additionally, rehabilitative training was found to promote neuronal activity and increase 5-HT-positive fibres at the lumbar enlargement, leading to significant improvements in motor functions. These findings highlight the potential of combinatorial therapies as a promising approach for treating chronic SCI.

While the study results are encouraging, it is important to acknowledge the limitations of the research. Firstly, the study was conducted in a rodent model, and therefore, caution should be exercised when extrapolating the findings to humans. Secondly, the study primarily focused on motor function and did not investigate sensory function or bladder control, which are also crucial outcomes in SCI. Thirdly, the long-term effects of the combined therapy remain unknown. Nonetheless, the results of this study are consistent with other preclinical research that has explored the potential of stem cell-based therapies for SCI treatment. Further investigations are required to optimise the therapeutic strategy and assess the long-term outcomes of stem cell-based therapies for SCI.

### Stem cell therapies with electronic acupuncture

Recently, an increased interest has been in investigating the potential benefits of combining electro-acupuncture (EA) with stem cell therapy for treating SCI. In a study published by Zhang and colleagues 2021, the effects of EA on the survival and migration of NSCs transplanted into injured rat spinal cords were investigated^58^. The results showed that the combination of EA and NSCs significantly improved NSC survival and migration in the injured spinal cords of rats, indicating its potential as a therapeutic approach for SCI.

Similarly, a study by Ding *et al.* ^59^ in 2009 examined the efficacy of Governor Vessel EA in promoting bone marrow MSC survival, neuroregeneration, and functional recovery in rat spinal cords. The study demonstrated that combination therapy effectively enhanced MSC survival and differentiation, resulting in functional recovery. These findings support electro-acupuncture and stem cell therapy as promising treatments for SCI.

## Clinical trials of stem cell-based combinatorial therapies for SCI

Clinical trials exploring stem cell-based combination therapies for SCI have been limited in number compared with preclinical studies (Table 4). This is primarily due to the complex nature of human SCI, which necessitates prioritising the safety and effectiveness of combination therapy over efficacy, a focus more commonly examined in preclinical trials. Additionally, standardised protocols to optimise outcomes in clinical trials are constrained by various limitations, while preclinical studies allow for greater flexibility. Various types of stem cells, including NSCs, ESCs, OSCs, iPSCs, and MSCs, have been studied in clinical trials. These cells are chosen based on their accessibility and lower risk of post-transplant rejection.

**TABLE 4:** Key preclinical studies on combinatorial therapies for SCI.

Author	Year	Modality	Result
Amr *et al.* ^60^	2014	Chitosan-laminin scaffold	Motor and sensory levels improved
Chen *et al.* ^61^	2020	NeuroRegen Scaffolds	Partial shallow sensory and autonomic nervous functional improvements
Zhao *et al.* ^62^	2017	NeuroRegen Scaffold	Expansion of sensation level and motor-evoked potential (MEP)-responsive area, increased finger activity, enhanced trunk stability, defecation sensation, and autonomic neural function recovery
Yoon *et al.* ^34^	2007	Granulocyte-macrophage-colony stimulating factor	Improved functional recovery
Zhu *et al.* ^64^	2016	Mononuclear Cell	Improved locomotor function

SCI, spinal cord injury.

### Clinical stem cell therapies with biomaterial scaffolds

In clinical trials investigating stem cell combination therapies for SCI, biomaterial scaffolds are commonly utilised, similar to their use in preclinical studies. One noteworthy case study involved the administration of peripheral nerve grafts combined with chitosan-laminin scaffolds and bone marrow-derived mesenchymal stem cells to 14 patients, resulting in improved motor levels, motor power, sensory level, and neurological level^60^. In a 3-year clinical trial, co-transplanting NeuroRegen scaffolds and autologous bone marrow mononuclear cells for repairing acute complete SCI led to functional improvement in patients concerning sensory levels, defecation sensation, physiological erection, superficial sensation, and deep sensation recovery^61^. An additional clinical trial involving eight patients investigated the effect of the combination of NeuroRegen scaffolds with human mesenchymal stem cells for repairing SCI, resulting in improved sensation levels, motor-evoked potential-responsive areas, motor activity of the forelimbs, balance, and autonomic neural function recovery^62^. These findings suggest that the use of biomaterial scaffolds in combination with stem cells may be a promising approach for treating SCI.

### Clinical Stem cell therapies with neurotrophic factors

Clinical trials investigating the use of stem cells in combination with neurotrophic factors such as Granulocyte-macrophage-colony factor, NGF, GDNF, and BDNF have been conducted. One notable clinical study focused on treating complete SCI in five patients using autologous bone marrow cell transplantation and granulocyte-macrophage-colony-stimulating factor^63^. The patients exhibited immediate sensory improvements, followed by motor and neurological function improvements after a few weeks. Similarly, a clinical trial on 35 patients in 2007 used autologous bone marrow cell transplantation and bone marrow stimulation with granulocyte-macrophage-colony stimulating factor^34^. During follow-up, neurological assessment, electrophysiological monitoring, and neuroimaging were conducted, revealing an increase in spinal cord diameter through MRI and no adverse effects of the operation on the patients.

A more recent study in 2016 assessed the safety and efficacy of umbilical cord blood mononuclear cell transplant therapy for chronic complete SCI in 28 patients^64^. Some patients received a 6x-week lithium carbonate injection course to stimulate neurotrophic factor secretion. Improvements were observed in the walking index of SCI and spinal cord independence measure scores, as well as significant improvements in autonomic neural functions through improved renal functions. These clinical trials provide evidence of the potential benefits of stem cell and neurotrophic factor combination therapies in treating SCI.

## Safety and ethical considerations

Stem cell-based combinatorial therapies have emerged as a promising approach to treatment, offering enhanced regenerative and reparative effects compared with single-cell therapies (Table 5). However, as with any medical intervention, careful consideration of potential risks and benefits is essential.

**TABLE 5:** Mechanisms of action of stem cells in SCI.

Mechanisms of action of stem cells in spinal cord injury	Description
Cell replacement	Stem cells differentiate into various cell types in the injured spinal cord, such as neurons, oligodendrocytes, and astrocytes, to replace lost or damaged cells and restore neural function.
Trophic support	Stem cells secrete various trophic factors, such as growth factors, cytokines, and extracellular vesicles, to promote cell survival, proliferation, differentiation, and axonal regeneration, and to reduce inflammation and apoptosis.
Immunomodulation	Stem cells modulate the immune response in the injured spinal cord by secreting anti-inflammatory factors, interacting with immune cells, and suppressing immune activation, to reduce tissue damage and promote tissue repair.
Angiogenesis	Stem cells induce angiogenesis in the injured spinal cord by secreting angiogenic factors and promoting the proliferation and differentiation of endothelial cells, to improve blood supply and tissue perfusion, and to enhance tissue repair and regeneration.

SCI, spinal cord injury.

Inflammation is a primary mechanism targeted by stem cell-based therapies for SCI^65^ (Table 6). MSCs have anti-inflammatory and immunomodulatory properties, making them a promising candidate for reducing inflammation in SCI. These cells can secrete various factors that modulate the immune response, reduce inflammation, and promote tissue repair. Moreover, they can stimulate the growth and survival of endogenous neural cells, thus enhancing the regenerative potential of the injured spinal cord. In animal models of SCI, NSCs have shown the ability to improve motor function and promote axon growth^66^. These cells may also play a role in remyelination, a crucial process for proper neural signalling and function.

**TABLE 6:** Stages of spinal cord injury and potential targets for stem cell-based therapies.

Stages of spinal cord injury and potential targets for stem cell-based therapies	Description
Acute inflammation	After injury, immune cells infiltrate the injury site, and pro-inflammatory cytokines, chemokines, and reactive oxygen species are released. Stem cells target acute inflammation by modulating immune cell activity, secreting anti-inflammatory factors, and scavenging reactive oxygen species.
Demyelination	The loss of myelin sheaths around axons impairs neuronal communication and function. Stem cells target demyelination by differentiating into oligodendrocytes and producing myelin, or by secreting trophic factors that stimulate remyelination.
Axonal degeneration	Axonal degeneration can occur due to mechanical damage, inflammation, or secondary cascades. Stem cells target axonal degeneration by promoting axonal regeneration through trophic support, guidance cues, and scaffolds.
Chronic inflammation	Chronic inflammation can lead to glial scar formation, fibrosis, and neuronal loss. Stem cells target chronic inflammation by suppressing immune activation, secreting anti-fibrotic factors, and promoting tissue repair and regeneration.
Neuronal loss	Neuronal loss can occur due to direct trauma, secondary injury, or chronic degeneration. Stem cells target neuronal loss by differentiating into neurons or by promoting neuronal survival and regeneration through trophic support, neuroprotection, and synaptic plasticity.

Biomaterials can also be employed with stem cells to provide structural support and promote the integration of transplanted cells. These materials create a favourable environment for stem cell survival, growth, and differentiation and provide physical support to the injured spinal cord, preventing further damage and promoting healing. Despite the promise of stem cell-based combinatorial therapies, potential risks, such as tumorigenicity, must be closely monitored. Biomaterials may also introduce the risk of infection or rejection. Therefore, rigorous safety testing and long-term monitoring are necessary to ensure the safety of these therapies.

Moreover, stem cell-based combinatorial therapies offer the potential for personalised treatments tailored to individual patients, which is particularly relevant for SCI, where the severity and location of the injury can vary widely. This multi-pronged approach targeting multiple mechanisms of injury and repair can improve outcomes and reduce the risk of adverse events. Customising stem cell-based therapies for SCI involves several considerations, including the type and source of stem cells used, imaging and diagnostic tools, comorbidities and individual factors, and potential associated risks. Different types of stem cells have unique properties and potential applications, and the source of stem cells may also impact the success of the treatment. Autologous stem cells may be less likely to be rejected by the immune system, while allogeneic stem cells may be more readily available and easier to manipulate. Imaging and diagnostic tools provide essential information about the extent and location of the injury, guiding the tailoring of stem cell-based therapies for each patient.

Careful consideration of any comorbidities or individual factors that affect the success of the treatment is critical. Patients with pre-existing medical conditions like diabetes or cardiovascular disease may require additional monitoring and support during stem cell-based therapies. Patients with autoimmune disorders or other medical conditions may require special precautions or adjustments to the treatment plan. Therefore, a personalised approach to treatment that considers each patient’s unique needs is necessary for the success of stem cell-based therapies in SCI.

Although stem cell-based combinatorial therapies hold immense promise for various neurological conditions, there are potential risks associated with their use. One significant risk is the potential for unwanted interactions between different cell types or therapies, which could result in unpredictable or adverse effects. Using biomaterials or supportive scaffolds can enhance integration and prevent unwanted interactions between different cell types. To minimise the risks associated with stem cell-based therapies, it is crucial to carefully select and characterise the stem cells used in these therapies and conduct rigorous safety testing before clinical use. Further research and development are necessary to optimise the safety and efficacy of these therapies. If successful, stem cell-based combinatorial therapies have the potential to provide a more personalised and effective approach to regenerative medicine.

Stem cell therapy for SCIs has sparked ethical concerns, particularly regarding the use of ESCs and the potential exploitation of vulnerable patients by unregulated for-profit clinics^67^. The ethical debate centres around the origin of ESCs, often obtained from discarded embryos after in-vitro fertilisation, raising questions about the moral status of the embryo and the ethical implications of their use in research and therapy^67^. The creation and disposal of human embryos for obtaining stem cells provoke ethical debates concerning the inception of life. On the other hand, iPSCs, derived from adult cells, introduce their own risks, including unintended differentiation, malignant transformation, reproductive cloning, and genetic engineering. Ethical challenges further arise for stem cell scientists and scholars regarding the oversight, regulation, and commercialisation of innovative stem cell technologies. Balancing scientific progress with ethical considerations becomes crucial when navigating the complex landscape of stem cell research. Moreover, the presence of unregulated for-profit clinics offering unproven stem cell treatments poses a risk of exploiting vulnerable patients in their quest for a cure. The term “unproven stem cell intervention industry” refers to a global market in which clinics directly offer stem cells, stem cell-derived components like exosomes, and non-stem cell-based cellular products to patients without sufficient scientific or clinical evidence^68^. This unregulated practice has resulted in numerous patient injuries and fatalities, posing a threat to legitimate research endeavours and undermining the regulatory authority responsible for safeguarding public health^68^. Addressing the issue of marketing unproven SCIs requires international coordination and cooperation, as it transcends national boundaries and no single country has effectively resolved it thus far. This underscores the importance of implementing rigorous regulatory standards and oversight to ensure ethical and responsible conduct in stem cell research and therapy.

Furthermore, concerns have been raised about the potential for unequal access to stem cell therapy, particularly in low-income or resource-limited settings^69^. The high cost of stem cell therapy and the lack of availability in certain regions could create disparities in access to treatment. This underscores the need for equitable distribution of stem cell therapies and consideration of the potential socioeconomic implications of these treatments. In addition, there are concerns about the possible non-therapeutic uses of stem cell therapy, such as cosmetic enhancement or performance enhancement. This raises questions about the appropriate use of stem cell therapies and the potential risks and benefits of using these treatments for non-therapeutic purposes.

The regulation of stem cell-based therapies presents an intricate and ever-evolving landscape, demanding a delicate equilibrium between patient accessibility, safety considerations, and treatment efficacy. While first-world countries, exemplified by the United States and the European Union, have established robust oversight mechanisms, developing nations showcase variable degrees of regulatory frameworks^70–72^. Governments must diligently update and refine their regulations, aligning them with the rapid strides of scientific progress. By doing so, the ethical and responsible integration of stem cell therapies into clinical practice can be ensured, ultimately benefiting patients and fostering the advancement of medical science.

## Challenges and future directions

Standardising protocols for isolating, characterising, and producing stem cells presents a significant obstacle to translating these therapies into clinical practice. Standardisation is essential to ensure consistency and safety in clinical trials and enable comparison of results across studies. Presently, there exists a wide variation in the methods used to isolate and characterise stem cells, which can compromise the quality of the stem cells and yield inconsistent results in clinical trials^73^. Significant efforts are underway to establish standardized protocols that define best practices and guidelines for the isolation, characterisation, and production of stem cells. These protocols aim to ensure consistency and reproducibility in stem cell research across various research institutions and laboratories. By implementing these standardized protocols, researchers can enhance the reliability and comparability of their findings. For example, in the field of iPSCs, the International Stem Cell Initiative (ISCI) developed guidelines for the characterisation and quality control of iPSC lines^74^. These guidelines provide researchers with a framework for assessing the pluripotency, genetic stability, and differentiation potential of iPSC lines, thereby ensuring the reliability of research outcomes. Similarly, in the field of MSCs, the International Society for Cellular Therapy (ISCT) established minimum criteria for defining and characterizing MSCs^75^. These criteria outline specific markers and functional properties that MSCs should possess, enabling researchers to accurately identify and work with MSC populations. Furthermore, establishing quality control measures, such as independent oversight committees, is equally important. These committees can ensure that clinical trials conform to ethical standards, monitor the quality and safety of stem cell-based therapies, and facilitate collaboration among research institutions. By overcoming these challenges, stem cell-based therapies have the potential to offer significant benefits to patients suffering from a variety of conditions.

Variations in stem cell isolation and characterisation methods can significantly impact the reproducibility of research results. Inconsistencies in methodologies pose a challenge when comparing and replicating findings across different studies, hindering scientific progress and the translation of stem cell therapies into clinical practice. The lack of standardized protocols for stem cell isolation and characterisation introduces variability in experimental procedures, making it difficult to establish reliable benchmarks and compare data effectively. Researchers may employ different techniques, reagents, culture conditions, and assessment criteria, resulting in disparate outcomes and conflicting conclusions. This lack of consistency and comparability not only hampers scientific advancement but also undermines the credibility and trustworthiness of stem cell research. It becomes challenging to validate and build upon previous studies, limiting the ability to develop robust evidence-based therapies.

Animal models accurately replicating human SCI are critical for successful translation. However, there are limitations to the available models, and accurately replicating the complex mechanisms of SCI in animals remains challenging. While animal models have been instrumental in understanding the underlying mechanisms of injury and developing potential therapies, they have limitations. They may not fully capture the complexity of human SCI. Researchers are developing more sophisticated animal models that better replicate human SCI, including genetically modified animals or new techniques to induce SCI that more accurately mimics human injury. Additionally, ex-vivo models, such as organoids, may provide a more accurate representation of the complexity of SCI. However, caution is necessary for extrapolating results from animal studies to human trials. Another significant challenge in stem cell-based therapies for SCI is the delivery of stem cells to the injury site. The success of stem cell therapy relies heavily on the delivery method, and the optimal mode of administration is still under debate. Current methods, including direct or intravenous injection into the spinal cord, have limitations. Furthermore, the survival and integration of transplanted stem cells into the injured tissue can be problematic, and biomaterials and scaffolds may provide a solution. These materials can help support stem cell survival and integration into the surrounding tissue and provide a suitable environment for cell differentiation and function. Overcoming these challenges is critical to ensure the safety and efficacy of stem cell-based therapies in human patients. Developing appropriate animal models that better replicate human SCI, optimising delivery methods, and enhancing stem cell survival and integration into the injured tissue will facilitate the translation of stem cell-based therapies for SCI into clinical practice.

Developing safe and effective stem cell-based therapies for SCI is a complex process that involves numerous challenges. One major obstacle is the risk of immune rejection when using allogeneic stem cells, which can limit therapy efficacy and cause serious adverse effects. Researchers are exploring strategies to minimise immune rejection, such as immunosuppressive drugs.

One promising avenue for future research is using innovative approaches, such as gene editing, to enhance the safety and efficacy of these therapies. Gene editing technologies like CRISPR-Cas9 can modify stem cells before transplantation to enhance their survival, integration, and differentiation in the injured tissue. This technology could also be used to develop more effective strategies for reducing the risk of immune rejection and other adverse effects associated with stem cell-based therapies. Finally, researchers are also exploring new methods for delivering stem cells to the injury site, which could improve therapy efficacy and outcomes. Ultimately, continued research in this field is essential to address the challenges of translating stem cell-based combinatorial therapies to clinical practice for SCI. Combinatorial therapies using stem cells hold promise for the treatment of SCIs. However, several challenges need to be addressed to maximise the effectiveness of stem cell-based combinatorial therapies for SCIs. Nevertheless, efforts and new directions are clearing these challenges gradually.

## Limitation of study

While this review provides valuable insights, it is important to acknowledge its limitations. One significant limitation is the exclusion of non-English language articles, which introduces a potential language bias and restricts the incorporation of relevant studies published in languages other than English. To address this limitation and ensure a more comprehensive understanding of the subject, future studies should consider including translations or conducting searches in multiple languages. This approach would allow for the integration of a wider range of perspectives leading to a more enriched and inclusive analysis.

## Conclusion

Stem cell-based combinatorial therapies show promise in the development of effective treatments for SCI. Combining stem cell therapy with other therapeutic approaches has enhanced spinal cord tissue regeneration and improved functional outcomes in preclinical studies. While challenges such as the need for protocol standardisation and appropriate animal models for testing exist, the potential benefits of these therapies are significant. Therefore, it is essential to conduct further research and development in this area to translate these therapies into effective treatments for patients with spinal cord injuries.

Innovative approaches such as gene editing and biomaterials can help overcome some of the challenges associated with stem cell therapy. Gene editing technologies such as CRISPR-Cas9 allow for precise modifications to the genome of cells, which can enhance stem cells’ survival, integration, and differentiation in the injured tissue. Furthermore, biomaterials and scaffolds can support the survival and integration of transplanted stem cells, providing a suitable environment for cell differentiation and function.

Clinical trials are necessary to assess these therapies’ safety and efficacy and ensure their successful translation into clinical practice. With continued advancements in our understanding of stem cells and their therapeutic potential, it is important to remain vigilant in our efforts to ensure safety and efficacy.

## Ethical approval

NA.

## Consent

NA.

## Source of funding

No external funding was received for this study.

## Author contribution

N.A.: conceptualization. Writing: all authors.

## Conflicts of interest disclosure

None.

## Research registration unique identifying number (UIN)

Not applicable.

## Guarantor

Nicholas Aderinto.

## Data availability statement

Data sharing is not applicable to this article.

## Provenance and peer review

Not commissioned, externally peer-reviewed.
